# Generalized joint hypermobility among school-aged children in Majmaah region, Saudi Arabia

**DOI:** 10.7717/peerj.9682

**Published:** 2020-08-11

**Authors:** Mohamed Sherif Sirajudeen, Mohamed Waly, Mazen Alqahtani, Msaad Alzhrani, Fahad Aldhafiri, Hariraja Muthusamy, Radhakrishnan Unnikrishnan, Rashmi Saibannavar, Wafa Alrubaia, Gopal Nambi

**Affiliations:** 1 Department of Physical Therapy and Health Rehabilitation, College of Applied Medical Sciences, Majmaah University, Majmaah, Saudi Arabia; 2 Department of Medical Equipment Technology, College of Applied Medical Sciences, Majmaah University, Majmaah, Saudi Arabia; 3 Department of Public Health, College of Applied Medical Sciences, Majmaah University, Majmaah, Saudi Arabia; 4 Department of Physical Therapy and Health Rehabilitation, College of Applied Medical Sciences, Prince Sattam Bin Abdul Aziz University, Alkharj, Saudi Arabia

**Keywords:** Beighton score, Joint hypermobility, Ligamentous laxity, Prevalence, Children, Saudi Arabia

## Abstract

Generalized joint hypermobility (GJH) is common among schoolchildren and usually benign. However, it may progressively lead to joint pain and developmental delay. Identifying GJH in school-aged children would facilitate the monitoring of early changes and planning for early rehabilitative intervention. Epidemiological studies addressing the prevalence of GJH among children in the Gulf region and Arab ethnicity are lacking. Hence, we aimed to determine the prevalence, pattern, and factors associated with GJH among school-aged children in the Majmaah region, Saudi Arabia. Male and female school-aged children 8–14 years of age from the Majmaah region of Saudi Arabia participated in this cross-sectional study. Beighton score was used to assess GJH. Personal characteristics such as age, height, weight, body mass index, and handedness were also collected. Descriptive statistics were obtained for personal characteristics, the point prevalence of hypermobility, frequency of Beighton score distribution, and prevalence of GJH. The associations between specific factors and the presence of GJH were analyzed using chi-square and Mann-whitney tests. Using the Beighton score cutoff ≥ 4 and ≥ 6, 15.2% and 7.6% of the school children in our study were diagnosed with GJH respectively. The prevalence of GJH was higher among females (16.8%) than among males (13.4%), but the difference was not statistically significant. The elbow joints (17.2%) were the most common hypermobile joints and the trunk (0.7%) was the least involved. The children with GJH were younger and had lesser BMI compared to children without GJH (*P* < 0.05). The prevalence reported in this study among school-aged children was comparable with those reported worldwide.

## Introduction

An increase in mobility of one or more joints compared to the normal range is referred to as joint hypermobility (Romeo et al., 2016). Ligament laxity is the primary cause of joint hypermobility (Grahame, 2000). Ligament laxity and the resulting joint hypermobility are cardinal features of genetic disorders such as Marfan syndrome, Ehlers-Danlos syndrome, or osteogenesis imperfecta *(Armon & Bale, 2012)*. However, in most cases, joint hypermobility is observed as a confined phenomenon referred to as generalized joint hypermobility (GJH). Rarely, in the absence of any genetic disorders, joint hypermobility is associated with features such as arthralgia, back pain, dislocation/subluxation, soft tissue rheumatic disorders, marfanoid habitus, skin abnormalities, eye signs, incompetence of the lower-limb vessel valves, or rectal hernia or prolapse and is termed joint hypermobility syndrome (Alsiri et al., 2020; Armon & Bale, 2012; Clinch et al., 2011; Palmer et al., 2014; Palmer et al., 2015).

The reported occurrence of GJH in children aged 6–15 years varies between 8.8% (Vougiouka, Moustaki & Tsanaktsi, 2000) and 64.6% (Lamari, Chueire & Cordeiro, 2005). The prevalence of GJH is high in girls and declines with age (Bird, Tribe & Bacon, 1978; Bulbena et al., 1992; Hudson et al., 1995; Larsson, Baum & Mudholkar, 1987; Silman, Day & Haskard, 1987). In a study of Swedish school children, Jansson et al. (2004) reported that girls presented with marked joint hypermobility versus boys regardless of age. However, the presentation of joint laxity in boys decreases as age increases, whereas joint laxity peaks in girls at 15 years of age. Earlier researchers demonstrated an influence of ethnic background on GJH; specifically, a high prevalence of GJH among Asian and African populations compared to the Western population (Beighton, Solomon & Soskolne, 1973; Bird, 2005; Carter & Wilkinson, 1964; Jessee, Owen Jr & Sagar, 1980). However, the prevalence of GJH in Arabic children is lacking in the literature (Sirajudeen, 2020).

Beighton score is a valid and reliable tool used worldwide to screen joint hypermobility (Juul et al., 2007; Smits-Engelsman, Klerks & Kirby, 2011). Beighton’s method includes the assessment of hypermobility in nine joints (bilateral thumbs, bilateral little fingers, bilateral elbows, bilateral knees, and trunk). The score ranges from 0 to 9; 1 point is awarded for the participant’s ability to perform each component of the test (Beighton, Solomon & Soskolne, 1973). All nine tests were easy to perform and provide quantitative data. Earlier prevalence studies adopted cutoffs ranging from ≥3 to ≥6 hypermobile joints for the diagnosis of GJH. The most frequent choice of cutoff score for GJH was ≥4 (Clinch et al., 2011). International Consortium on the Ehlers Danlos syndromes (EDS) proposed cut-off score of ≥6 for diagnosis of GJH for pre-pubertal children and adolescents (Malfait et al., 2017; Reuter & Fichthorn, 2019). A recent systematic review on Measurement Properties of Clinical Assessment Methods for Classifying Generalized Joint Hypermobility also recommended a cut-off of minimum ≥6 for diagnosis of GJH in children (Juul-Kristensen et al., 2017). Juul et al. (2007) and Smits-Engelsman, Klerks & Kirby (2011) recommended the administration of standard protocols in children.

Prevalence studies related to GJH cater vital data about the burden of this entity in each population reflected by number of individuals affected. This provide valuable input to plan the healthcare needs. GJH is common among schoolchildren and usually benign. However, it may lead to joint pain and developmental delay (Jaffe et al., 1988). Identifying GJH during the school years would facilitate the monitoring of early changes and planning for early rehabilitative intervention (Lamari, Chueire & Cordeiro, 2005). Epidemiological studies addressing the prevalence of GJH among children reflecting Arab ethnicity in the Gulf region are lacking (Sirajudeen, 2020). Hence, this study, the first of its kind in Saudi Arabia and the entire Gulf region, aimed to explore the prevalence of GJH and its associated factors in school-aged children in the Majmaah region.

## Methods

### Study design, setting, and participants

Male and female school-aged children aged 8–14 years from the Majmaah region of Saudi Arabia participated in this cross-sectional study. Permission was obtained from the Information and Planning Authority for Education Majmaah, under the Ministry of Education, Saudi Arabia. Majmaah region consists of 15 primary schools with 1755 children belonging to the 8 -14 age group. Out of the which, 3 schools were selected by cluster sampling method and all the 311 children were approached for the study. A letter describing the study design and seeking the cooperation of Headteachers of Schools was sent. The data were collected between October and December 2018 in their respective schools. Children with any apparent or reported disabilities such as cognitive, developmental, or bodily as per the medical data available in school were not included in this study (Romeo et al., 2016).

### Anthropometry measurements

Data related to personal characteristics like age (years), sex, height (centimeters), and weight (kilograms) were collected. Height and weight were measured by the gold standard method (in bare feet; measured to the closest one cm and 100 g, respectively). The body mass index (BMI) calculation was performed using a metric formula, weight (in kilograms) divided by height (in meters squared) and children were sorted as underweight (BMI < 18.5), ideal weight (BMI 18.5–24.9), overweight (BMI 25–29.9), or obese (BMI > 30) as per the recommended criteria (Gerver & de Bruin, 2001).

### Beighton score for screening GJH

The screening criteria comprises five clinical tests; each was assigned 0 or 1 based on the subject’s ability to complete it. These test scores are summed at the end, and the totals range from 0 to 9.

1. Researcher passively performs thumb apposition to the flexor side of the forearm on the right and left sides, and a score of 1 is awarded if the entire thumb touches the flexor side of the forearm (90° of shoulder flexion with elbow extension and forearm pronation);
2. Researcher passively performs dorsiflexion of the fifth metacarpophalangeal joint on the right and left sides ≥90° (sitting on chair, arm abducted to 80°, elbow flexed to 90°, forearm resting on the table in pronation);
3. Researcher passively performs hyperextension of the elbow joint on the right and left sides ≥10° (with subject on a chair with shoulder positioned to 90° of flexion with forearm supinated);
4. Researcher passively performs hyperextension of the knee on the right and left sides ≥ 10° (subject positioned supine lying with legs supported on a table); and
5. Subjects asked to flex the trunk with knees in the extended position so that palms rest easily on the ground (Smits-Engelsman, Klerks & Kirby, 2011).

The cutoff score ≥4 identified GJH (Clinch et al., 2011). A cutoff score ≥6 was used to analyze any association. A 360° universal goniometer was used to measure joint angles. The measurements and Beighton maneuvers were performed by two physical therapists with 15 years of experience in the pediatric practice and research. The inter-rater reliability of the therapists was measured in 20 subjects and determined as 0.98 (Intraclass correlation).

### Sample size calculation

We calculated sample size using the Sample Size Calculation for Estimating a Single Proportion method. Since this is the first study to investigate the prevalence of GJH in the entire Gulf region, we used the prevalence of 16% reported among Egypt children (El-Garf, Mahmoud & Mahgoub, 1998). The required sample size was 207 with 95% confidence and 5% absolute precision.

### Ethical consideration

Approval was obtained from Majmaah University Ethical Committee (no. MUREC –Oct21/COM-2018/6). Each child’s parents were provided information about the study, after which they provided a written informed consent to permit their child to participate in the research. Moreover, assent was also obtained from the participating children before their enrollment in this study.

### Statistical analysis

The data were recorded in a Microsoft Excel spreadsheet and analyzed using SPSS (version 17.0) for Windows. Descriptive statistics were obtained for personal characteristics, the point prevalence of hypermobility, and frequency of Beighton score distribution. The prevalence of GJH was calculated by dividing the number of children diagnosed with GJH (Beighton cutoff score ≥4 or ≥6) by the total number of students who participated in the study. The data were tested for normality and homogeneity of variance for deciding appropriate statistics. The chi-square statistic was used to compute the association between binary variables and the presence/absence of GJH. The Mann-whitney test was used to determine association between the continuous variables and the presence/absence of GJH. The level of probability of 5% was used to indicate statistical significance.

## Results

Out of 311 children approached, eight were sick on the day of examination. A total of 303 children participated in this study. Their personal characteristics are presented in Table 1. Most of the participating children were female (53.1%). The mean age of the children was 10.74 years and ranged from 8 to 14 years. Most of the children were underweight (53.1%), whereas 34.7%, 7.9%, and 4.3% were normal weight, overweight, and obese, respectively. Most of the participants were right-handed (95.7%).

**Table 1:** Personal characteristics of participating children.

**Characteristic**	**Frequency (%)/Mean (SD)**
***Sex***	
Male	142 (46.9)
Female	161 (53.1)
***Age (years)***	10.74 (1.24)
***Height***	138.10 (11.7)
***Weight***	37.90 (12.1)
***BMI***	
Underweight	161 (53.1)
Normal weight	105 (34.7)
Overweight	24 (7.9)
Obese	13 (4.3)
***Hand dominance***	
Left	13 (4.3)
Right	290 (95.7)

**Notes.**

Abbreviations BMI body mass index SD standard deviation

The distribution of the total Beighton score of the participants is presented in Table 2. Most of the participants (75.6%) did not exhibit hypermobility in any of the tested joints (Beighton score, 0). None of the participants in this present study demonstrated hypermobility in all tested sites. The occurrence of GJH as formulated by a Beighton cutoff score ≥4 in the 303 participated primary school children was 15.2% (males, 13.4%; and females, 16.8%). When a more vigorous cutoff (≥6) was used, the prevalence was 7.6% (males, 4.5%; and females, 9.9%).

**Table 2:** Frequency distribution of total Beighton score and prevalence of generalized joint hypermobility (based on cutoff ≥4 or ≥6).

**Score**	**Frequency (%)**
***0***	229 (75.6)
1	5 (1.7)
2	22 (7.3)
3	1 (0.3)
***4***	21 (6.9)
5	2 (0.7)
6	13 (4.3)
7	1 (0.3)
***8***	9 (3)
9	0 (0)
**Hypermobility**	
Cutoff ≥4	46 (15.2)
Cutoff ≥6	23 (7.6)

Table 3 represents the distribution of the participants’ joint hypermobility. The occurrence of hypermobility was high in the elbows (17.2%), followed by the thumbs (12.5%), little fingers (12.5%), knees (8.6%), and trunk (0.7%). The proportion of males and females with hypermobility of left little finger (15.5% in males vs. 8.1% in females), right little finger (18.3% in males vs. 7.4% in females), left thumb (4.9% in males vs. 19.2% in females) and right thumb (4.9% in males vs. 18% in females) were statistically significant (*P* < 0.05). Trunk hypermobility in males was unusual, as none of our 142 male subjects could place their palms flat on the ground with the knees in full extension.

**Table 3:** Point prevalence of joint hypermobility at various sites used in the Beighton criteria (Cut-off ≥4).

Beighton site	All (*n* = 303) Frequency (%)	Male (*n* = 142) Frequency (%)	Female (*n* = 161) Frequency (%)	*P* value^a^
**Little Finger**				
Left	35 (11.5)	22 (15.5)	13 (8.1)	0.043^*^
Right	38 (12.5)	26 (18.3)	12 (7.4)	0.004^*^
**Thump**				
Left	38 (12.5)	7 (4.9)	31 (19.2)	0.000^*^
Right	36 (11.9)	7 (4.9)	29 (18)	0.002^*^
**Elbow**				
Left	52 (17.2)	25 (17.6)	27 (16.8)	0.618
Right	52 (17.2)	25 (17.6)	27 (16.8)	0.636
**Knee**				
Left	26 (8.6)	9 (6.3)	17 (10.6)	0.191
Right	24 (7.9)	8 (5.6)	16 (9.9)	0.166
**Trunk**	2 (0.7)	0 (0)	2 (1.2)	0.182

**Notes.**

a *P* Value for Chi square test.

* Statistically significant *P* < 0.05.

The association between personal characteristics and GJH is summarized in Table 4. The prevalence of GJH was higher among females (16.8%) than males (13.4%), but this difference was not statistically significant. The children with GJH (Cut-off ≥4) were younger compared to children without GJH. The difference in age was statistically significant (*P* < 0.05) for the entire sample and marginally significant (*P* = 0.05) in males but not in females. The BMI of children with GJH was lesser compared to children without GJH. The difference in BMI was statistically significant (*P* < 0.05) for the entire sample (Cut-off ≥4) and in females (Cut-off ≥4 and ≥6) but not in males. The children with GJH were similar to children without GJH with regard to hand dominance.

**Table 4:** Association between personal characteristics and presence of generalized joint hypermobility (based on Beighton score ≥4 , or ≥6).

**Characteristics**	**Hypermobility (Beighton score ≥4)**			**Hypermobility (Beighton score ≥6)**		
	**Yes**	**No**	***P* value**	**Yes**	**No**	***P* value**
***Total (*N* = 303*)****						
***Sex***			0.411^a^			0.100^a^
Male	19	123		7	135	
Female	27	134		16	145	
***Age (years) ; Mean (SD)***	10.39 (1.10)	10.79 (1.25)	0.025^b^^*^	10.34 (1.33)	10.76 (1.22)	0.167^b^
***BMI; Mean (SD)***	17.87 (3.64)	19.71 (4.97)	0.029^b^^*^	17.64 (2.97)	19.58 (4.93)	0.090^b^
***Hand dominance***			0.441^a^			0.291^a^
Left	3	12		2	13	
Right	43	245		21	267	
***Males (N = 142)***						
***Age (years); Mean (SD)***	10.69 (0.83)	11.04 (1.23)	0.055^b^	11 (0.58)	10.99 (1.21)	0.694^b^
***BMI; Mean (SD)***	18.72 (3.84)	19.87 (5.46)	0.75^b^	18.75 (2.91)	19.77 (5.37)	0.973^b^
***Hand dominance***			0.745^a^			0.455^a^
Left	2	9		1	10	
Right	17	114		6	125	
***Females (N = 161)***						
***Age (years); Mean (SD)***	10.19 (1.25)	10.59 (1.25)	0.146^b^	10.07 (1.49)	10.57 (1.22)	0.163^b^
***BMI; Mean (SD)***	17.28 (3.44)	19.57 (4.50)	0.011^b^^*^	17.15 (2.95)	19.41 (4.50)	0.041^b^^*^
***Hand dominance***			0.433^a^			0.561^a^
Left	1	3		1	3	
Right	26	131		15	142	

**Notes.**

BMI Body mass index SD Standard deviation

a *P* Value for Chi-square test.

b *P* Value for Mann–Whitney Test.

* Statistically significant (*P* < 0.05).

## Discussion

This is the first study in the entire Gulf region, reflecting the Arab ethnicity, to report the prevalence of GJH among school children aged 8–14 years. In the present study, the prevalence of GJH was 15.2% with use of the cutoff score ≥4 hypermobile joints from the 9-point Beighton scoring system. The prevalence reported in our study is comparable to those reported of children in Denmark (9.4%), Egypt (16%), and the United Kingdom (19.2%) but lower than those reported of children in Iceland (27.7%), Italy (35.4%), and India (61.2%) (Clinch et al., 2011; El-Garf, Mahmoud & Mahgoub, 1998; Gyldenkerne et al., 2007; Hasija, Khubchandani & Shenoi, 2008; Leone et al., 2009; Qvindesland & Jonsson, 1999). The difference in the prevalence of GJH reported in this study as compared to some other locations may be attributed to the ethnic influence. Earlier researchers also demonstrated an influence of ethnic background on GJH; specifically, a high prevalence of GJH among Asian and African populations compared to the Western population (Beighton, Solomon & Soskolne, 1973; Bird, 2005; Carter & Wilkinson, 1964; Jessee, Owen Jr & Sagar, 1980). The prevalence of GJH was higher among females (16.8%) than males (13.4%), but this difference was not statistically significant (P <0.05). This finding is consistent with the rates reported by El-Garf et al. of Egyptian children (males, 14.4%; females, 18%) (El-Garf, Mahmoud & Mahgoub, 1998). Gyldenkerne et al. reported a significantly higher occurrence of GJH among female children (16.6%) than among male children (3%) in Denmark (Gyldenkerne et al., 2007). None of the earlier studies reported a higher incidence of GJH in males than in females (Clinch et al., 2011; El-Garf, Mahmoud & Mahgoub, 1998; Gyldenkerne et al., 2007; Hasija, Khubchandani & Shenoi, 2008; Leone et al., 2009; Qvindesland & Jonsson, 1999).

The female children in our study predominantly presented with hypermobility of the thumb and elbow, whereas the male children were commonly presented with hypermobility at the fingers and elbows. It was fascinating that our subjects showed considerably less hypermobility in the lumbar spine. This may be described by the factuality that the most of the range of flexion of the lumbar spine is a conjoint of extensibility of the hamstrings and actual vertebral flexion (Corben, Lewis & Petty, 2008); short hamstrings would be a factors that are associated with reduced lumbar flexion in males (Gajdosik, Albert & Mitman, 1994). A perceived reduction in flexion of the lumbar spine may have been caused by tight hamstrings, which in turn could be a reason for the low occurrence of hypermobility of the lumbar spine among the boys in the present study.

In our study, the rate of hypermobility decreased as age increased in male but not female subjects. Studies of Egyptian and Swedish children reported an inverse age-related decrease in the prevalence of GJH in both males and females (El-Garf, Mahmoud & Mahgoub, 1998; Jansson et al., 2004)*.* The degree of collagen cross-linking is related to joint hypermobility, which is believed to increase the collagen’s ability to attract and hold water and thereby increase joint mobility. As age increases, body water decreases and cross-linking of collagen molecules increases, accounting for the decrease in joint hypermobility. Increased muscle fiber diameter is another factor that would reduce joint mobility at an older age (Lamari, Chueire & Cordeiro, 2005; Smits-Engelsman, Klerks & Kirby, 2011). Beighton et al. stated that joint laxity is at its maximum at birth and decreases quickly during childhood, less quickly in adolescence, and more slowly during adulthood (Beighton, Grahame & Bird, 1999).

In our study, the prevalence of GJH was positively associated with BMI in females but not in males. The BMI of female children with GJH was lesser compared to those without GJH. Clinch et al. also reported a positive association between GJH and BMI among females but not males. But in their study, obese females were 2.7 times more likely to be hypermobile than underweight females (Clinch et al., 2011). In contrast, a previous study from India reported an association between hypermobility and moderate to severe malnutrition (Hasija, Khubchandani & Shenoi, 2008). Using cut-off ≥6, the prevalence of GJH was associated with BMI in females and not in males. No other associations were seen using cut-off ≥6 to define GJH. A previous study in the United Kingdom using a Beighton score cutoff ≥6 compared with ≥4 to measure generalized joint laxity (GJL), reported stronger evidence of associations between physical activity and maternal education. The authors also suggested raising the cutoff from ≥4 to ≥6 to determine GJH (Clinch et al., 2011). We also recommend the use of criteria proposed by International Consortium on the EDS; Cut-off ≥6 for prepubertal children and adolescents to define GJH in the future studies *(Malfait et al., 2017)*. This will hopefully facilitate comparison between the results and reduces the ambiguity in the future*.*

### Study limitations

One of the limitations of the present study is that the clinical symptoms studied by previous researchers were not assessed (El-Garf, Mahmoud & Mahgoub, 1998; Hasija, Khubchandani & Shenoi, 2008; Leone et al., 2009; Qvindesland & Jonsson, 1999; Seçkin et al., 2005). Thus, the correlations between GJH and clinical symptoms are unclear. Al-Rawi, Al-Aszawi & Al-Chalabi (1985) who studied university students aged 20–24 years, reported a correlation between joint hypermobility and symptoms/signs including joint complaints and ligamentous sprains. Mikkelson and Qvindesland studied 12-year-old children and reported that hypermobility was not associated with any kind of joint symptoms (Grahame, 1999; Mikkelsson, Salminen & Kautiainen, 1996; Qvindesland & Jonsson, 1999). A further limitation of the present study is that parental or sibling hypermobility, which could be an added risk factor for hypermobility, was not assessed. Like the limitations encountered in any other observational studies, we can neither rule out confounders and chance nor establish causal or temporal relationships of the reported associations.

## Conclusion

Using the Beighton score cutoff ≥4 and ≥6, 15.2% and 7.6% of the school children in our study were diagnosed with GJH respectively. The prevalence reported in this study among school-aged children was comparable with those reported worldwide. The elbow joints were the most common hypermobile joints and the trunk was the least involved. The children with GJH were younger and had lesser BMI compared to children without GJH.

## Supplemental Information

10.7717/peerj.9682/supp-1 Supplemental Information 1 Raw data and Coding

## Additional Information and Declarations

### Data Availability

The following information was supplied regarding data availability: The raw measurements are available in the Supplementary File.

## References

[ref-1] Al-Rawi ZS, Al-Aszawi AJ, Al-Chalabi T (1985). Joint mobility among university students in Iraq. British Journal of Rheumatology.

[ref-2] Alsiri N, Cramp M, Barnett S, Palmer S (2020). Gait biomechanics in joint hypermobility syndrome: a spatiotemporal, kinematic and kinetic analysis. Musculoskeletal Care.

[ref-3] Armon K, Bale P (2012). Identifying heritable connective tissue disorders in childhood. The Practitioner.

[ref-4] Beighton P, Grahame R, Bird H (1999). Hypermobility of joints.

[ref-5] Beighton P, Solomon L, Soskolne CL (1973). Articular mobility in an African population. Annals of the Rheumatic Diseases.

[ref-6] Bird HA (2005). Joint hypermobility in children. Rheumatology.

[ref-7] Bird HA, Tribe CR, Bacon PA (1978). Joint hypermobility leading to osteoarthrosis and chondrocalcinosis. Annals of the Rheumatic Diseases.

[ref-8] Bulbena A, Duro JC, Porta M, Faus S, Vallescar R (1992). Clinical assessment of hypermobility of joints: assembling criteria. Journal of Rheumatology.

[ref-9] Carter C, Wilkinson J (1964). Persistent joint laxity and congenital dislocation of the hip. Journal of Bone and Joint Surgery. British Volume.

[ref-10] Clinch J, Deere K, Sayers A, Palmer S, Riddoch C, Tobias JH, Clark EM (2011). Epidemiology of generalized joint laxity (hypermobility) in fourteen-year-old children from the UK: a population-based evaluation. Arthritis Rheumatism.

[ref-11] Corben T, Lewis JS, Petty NJ (2008). Contribution of lumbar spine and hip movements during the palms to floor test in individuals with diagnosed hypermobility syndrome. Physiotherapy Theory and Practice.

[ref-12] El-Garf AK, Mahmoud GA, Mahgoub EH (1998). Hypermobility among Egyptian children: prevalence and features. Journal of Rheumatology.

[ref-13] Gajdosik RL, Albert CR, Mitman JJ (1994). Influence of hamstring length on the standing position and flexion range of motion of the pelvic angle, lumbar angle and thoracic angle. Journal of Orthopaedic and Sports Physical Therapy.

[ref-14] Gerver WJM, de Bruin R (2001). Paediatric morphometrics: a reference manual.

[ref-15] Grahame R (1999). Joint hypermobility and genetic collagen disorders: are they related?. Archives of Disease in Childhood.

[ref-16] Grahame R (2000). Hypermobility—not a circus act. International Journal of Clinical Practice.

[ref-17] Gyldenkerne B, Iversen K, Roegind H, Fastrup D, Hall K, Remvig L (2007). Prevalence of general hypermobility in 12–13-year-old school children and impact of an intervention against injury and pain incidence. Advances in Physiotherapy.

[ref-18] Hasija RP, Khubchandani RP, Shenoi S (2008). Pediatric rheumatology-joint hypermobility in Indian children. Clinical and Experimental Rheumatology.

[ref-19] Hudson N, Starr MR, Esdaile JM, Fitzcharles MA (1995). Diagnostic associations with hypermobility in rheumatology patients. British Journal of Rheumatology.

[ref-20] Jaffe M, Tirosh E, Cohen A, Taub Y (1988). Joint mobility and motor development. Archives of disease in childhood.

[ref-21] Jansson A, Saartok T, Werner S, Renström P (2004). General joint laxity in 1845 Swedish school children of different ages: age- and gender-specific distributions. Acta Pæ diatrica.

[ref-22] Jessee EF, Owen Jr DS, Sagar KB (1980). The benign hypermobile joint syndrome. Arthtitis and Rheumatism.

[ref-23] Juul B, Rogind H, Jensen DV, Remvig L (2007). Inter-examiner reproducibility of tests and criteria for generalized joint hypermobility and benign joint hypermobility syndrome. Rheumatology.

[ref-24] Juul-Kristensen B, Schmedling K, Rombaut L, Lund H, Engelbert RHH (2017). Measurement properties of clinical assessment methods for classifying generalized joint hypermobility—A systematic review. American Journal of Medical Genetics Part C: Seminars in Medical Genetics.

[ref-25] Lamari NM, Chueire AG, Cordeiro JA (2005). Analysis of joint mobility patterns among preschool children. Sao Paulo Medical Journal.

[ref-26] Larsson LG, Baum J, Mudholkar GS (1987). Hypermobility: features and differential incidence between the sexes. Arthtitis and Rheumatism.

[ref-27] Leone V, Tornese G, Zerial M, Locatelli C, Ciambra R, Bensa M, Pocecco M (2009). Joint hypermobility and its relationship to musculoskeletal pain in schoolchildren: a cross-sectional study. Archives of Disease in Childhood.

[ref-28] Malfait F, Francomano C, Byers P, Belmont J, Berglund B, Black J, Bloom L, Bowen JM, Brady AF, Burrows NP, Castori M, Cohen H, Colombi M, Demirdas S, Backer JD, Paepe AD, Fournel-Gigleux S, Frank M, Ghali N, Giunta C, Grahame R, Hakim A, Jeunemaitre X, Johnson D, Juul-Kristensen B, Kapferer-Seebacher I, Kazkaz H, Kosho T, Lavallee ME, Levy H, Mendoza-Londono R, Pepin M, Pope FM, Reinstein E, Robert L, Rohrbach M, Sanders L, Sobey GJ, Damme TV, Vandersteen A, Mourik CV, Voermans N, Wheeldon N, Zschocke J, Tinkl B (2017). The 2017 international classification of the Ehlers–Danlos syndromes. American Journal of Medical Genetics Part C: Seminars in Medical Genetics.

[ref-29] Mikkelsson M, Salminen JJ, Kautiainen H (1996). Joint hypermobility is not a contributing factor to musculoskeletal pain in pre-adolescents. Journal of Rheumatology.

[ref-30] Palmer S, Bailey S, Barker L, Barney L, Elliott A (2014). The effectiveness of therapeutic exercise for joint hypermobility syndrome: a systematic review. Physiotherapy.

[ref-31] Palmer S, Cramp F, Lewis R, Muhammad S, Clark E (2015). Diagnosis, management and assessment of adults with joint hypermobility syndrome: a UK-wide survey of physiotherapy practice. Musculoskeletal Care.

[ref-32] Qvindesland A, Jonsson H (1999). Articular hypermobility in Icelandic 12-year-olds. Rheumatology.

[ref-33] Reuter PR, Fichthorn KR (2019). Prevalence of generalized joint hypermobility, musculoskeletal injuries, and chronic musculoskeletal pain among American university students. PeerJ.

[ref-34] Romeo DM, Lucibello S, Musto E, Brogna C, Ferrantini G, Velli C, Cota F, Ricci D, Mercuri E (2016). Assessing joint hypermobility in preschool-aged children. Jornal de Pediatria.

[ref-35] Seçkin Ü, Tur BS, Ö Yılmaz, I Yağcı, Bodur H, Arasıl T (2005). The prevalence of joint hypermobility among high school students. Rheumatology International.

[ref-36] Silman AJ, Day SJ, Haskard DO (1987). Factors associated with joint mobility in an adolescent population. Annals of the Rheumatic Diseases.

[ref-37] Sirajudeen MS (2020). Physical therapy management for child with generalized joint hypermobility. Majmaah Journal of Health Sciences.

[ref-38] Smits-Engelsman B, Klerks M, Kirby A (2011). Beighton score: a valid measure for generalized hypermobility in children. Jornal de Pediatria.

[ref-39] Vougiouka O, Moustaki M, Tsanaktsi M (2000). Benign hypermobility syndrome in Greek schoolchildren. European Journal of Pediatrics.

